# An early Phase II randomised controlled trial testing the effect on persecutory delusions of using CBT to reduce negative cognitions about the self: The potential benefits of enhancing self confidence

**DOI:** 10.1016/j.schres.2014.10.038

**Published:** 2014-12

**Authors:** Daniel Freeman, Katherine Pugh, Graham Dunn, Nicole Evans, Bryony Sheaves, Felicity Waite, Emma Černis, Rachel Lister, David Fowler

**Affiliations:** a Department of Psychiatry, University of Oxford, UK; b Sussex Partnership NHS Foundation Trust, UK; c Centre for Biostatistics, Institute of Population Health, University of Manchester, UK; d MRC NW Hub for Trials Methodology Research, UK; e Department of Psychology, University of Sussex, UK

**Keywords:** Paranoia, Delusions, Self-esteem, Well-being, Clinical trial

## Abstract

**Background:**

Research has shown that paranoia may directly build on negative ideas about the self. Feeling inferior can lead to ideas of vulnerability. The clinical prediction is that decreasing negative self cognitions will reduce paranoia.

**Method:**

Thirty patients with persistent persecutory delusions were randomised to receive brief CBT in addition to standard care or to standard care (ISRCTN06118265). The six session intervention was designed to decrease negative, and increase positive, self cognitions. Assessments at baseline, 8 weeks (posttreatment) and 12 weeks were carried out by a rater blind to allocation. The primary outcomes were posttreatment scores for negative self beliefs and paranoia. Secondary outcomes were psychological well-being, positive beliefs about the self, persecutory delusions, social comparison, self-esteem, anxiety, and depression.

**Results:**

Trial recruitment and retention were feasible and the intervention highly acceptable to the patients. All patients provided follow-up data. Posttreatment there was a small reduction in negative self beliefs (Cohen's *d* = 0.24) and a moderate reduction in paranoia (*d* = 0.59), but these were not statistically significant. There were statistically significant improvements in psychological well-being (*d* = 1.16), positive beliefs about the self (*d* = 1.00), negative social comparison (*d* = 0.88), self-esteem (*d* = 0.62), and depression (*d* = 0.68). No improvements were maintained. No adverse events were associated with the intervention.

**Conclusions:**

The intervention produced short-term gains consistent with the prediction that improving cognitions about the self will reduce persecutory delusions. The improvement in psychological well-being is important in its own right. We recommend that the different elements of the intervention are tested separately and that the treatment is lengthened.

## 1 Introduction

Persecutory delusions are one of the key psychotic experiences. Existing treatments for psychotic experiences, pharmacological and psychological, are only partially effective (Leucht et al., 2013, van der Gaag et al., 2014). Our approach to developing a much more efficacious treatment is to target the main mechanisms causing the delusions. We take a step by step approach to translation of the advances in understanding persecutory delusions (Freeman and Garety, 2014), based upon an interventionist-causal model approach (Kendler and Campbell, 2009). One of the putative causal factors with the greatest empirical support is negative beliefs about the self (Freeman et al., 2002, Kesting and Lincoln, 2013, Tiernan et al., 2014). Therefore in the current study we aimed to reduce negative thoughts about the self in order to test the effect on clinical paranoia.

**Fig. 1:**
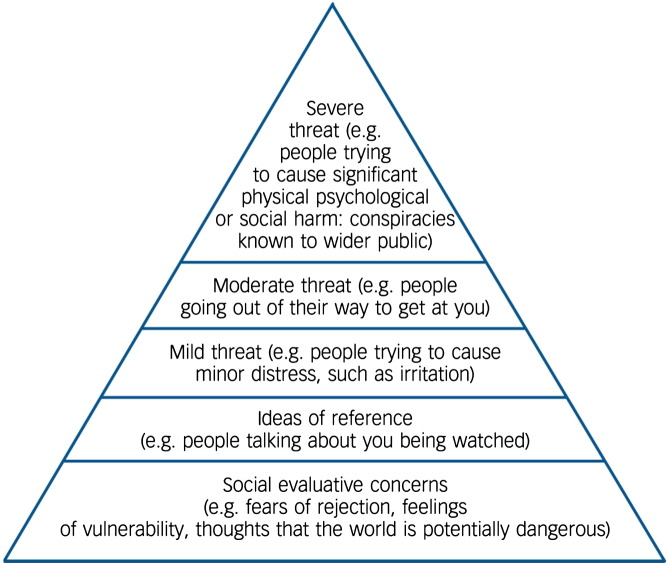
The paranoia hierarchy (Freeman et al., 2005).

Negative thoughts about the self can lead to feelings of being different, apart, inferior and hence vulnerable, a perspective central to the theoretical conceptualisation of the paranoia hierarchy (see Fig. 1) (Freeman et al., 2005). Paranoia will flourish when an individual perceives him or herself as vulnerable. Three recent systematic reviews have established that paranoia is associated with negative cognitions about the self (Garety and Freeman, 2013, Kesting and Lincoln, 2013, Tiernan et al., 2014). There is also longitudinal and experimental support. In a study of 301 patients with psychosis assessed three times over 12 months, negative cognitions about the self predicted the persistence of persecutory delusions, with little evidence of reverse causation (Fowler et al., 2012). A study of sixty patients with persecutory delusions showed that negative beliefs about the self predicted the persistence of paranoia over the next six months (Vorontsova et al., 2013). In an experimental study using virtual reality it was shown that increasing negative views of the self leads to an increase in paranoia in vulnerable individuals (Freeman et al., 2014a). There are numerous other reports of links between negative self-concepts and paranoia (Bentall et al., 2009, Hutton et al., 2013, Lincoln et al., 2013, Atherton et al., in press). Adverse experiences and environments will have a major contribution to the development of negative self-concepts (e.g. Selten and Cantor-Graae, 2005, Gracie et al., 2007, Bentall et al., 2012).

The clinical implication is that reducing negative thoughts about the self will lead to reductions in persecutory delusions. Consistent with this, self-esteem interventions for patients with psychosis have also been shown to lead to improvements in positive symptoms (e.g. Lecomte et al., 1999, Hall and Tarrier, 2003). These studies have not examined persecutory delusions specifically. We set-out to reduce the short-term negative self-cognitions in patients with persistent persecutory delusions. Our view is that concepts such as negative self cognitions, self-esteem, and negative social comparison are closely related phenomena, tied to levels of depression. Therefore we measured all of these concepts. The evaluation was an early Phase II clinical trial, but with similarities to an experimental test. We used both standard CBT techniques for negative thoughts but also techniques from positive psychology to increase positive thoughts about the self (Freeman and Freeman, 2012); this was on the basis of clinical experience and research that shows that negative and positive affect are inversely correlated and overlap in genetic and environmental causes (e.g. Kendler et al., 2011, Nes et al., 2013). That is, our approach was to try to diminish negative thoughts and build positive thoughts. This is consistent with Woods and Tarrier's (2010) argument that ‘positive and negative characteristics cannot logically be studied or changed in isolation’. The primary hypothesis was that a brief intervention focused on reducing negative beliefs about the self would immediately post-treatment produce reductions both in negative beliefs and in paranoia. The secondary prediction was that there would be improvements in related variables: positive beliefs about the self, comparison of self to others, self esteem, well-being, anxiety, and depression. Of course improvements in well-being and depression in this group would be valuable irrespective of effects on paranoia; almost half of patients with persecutory delusions have well-being scores in the lowest 2% of the population (Freeman et al., 2014b) and a similar proportion meet diagnostic criteria for depression (Vorontsova et al., 2013). We also examined whether any improvements due to treatment would be maintained at a follow-up assessment, although at this stage of intervention development we were focussed upon immediate change with treatment.

## 2 Method

### 2.1 Participants

**Fig. 2:**
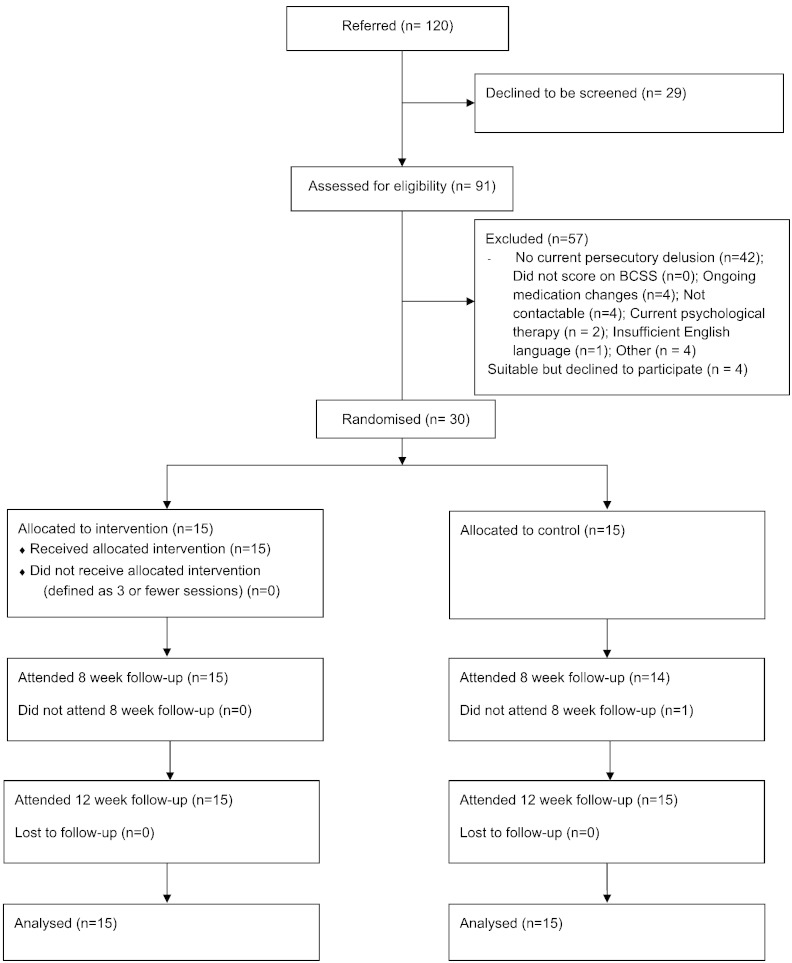
Flow diagram of patient recruitment and trial progress.

30 patients were recruited from Oxford Health NHS Foundation Trust. The inclusion criteria were: a current persecutory delusion as defined by Freeman and Garety (2000); scoring at least 3 on the conviction scale of the PSYRATS (Haddock et al., 1999); the delusion had persisted for at least three months; a clinical diagnosis of schizophrenia, schizoaffective disorder or delusional disorder (i.e. a diagnosis of non-affective psychosis); negative beliefs about the self as indicated by endorsing at least one negative schematic belief on the Brief Core Schema Scale (BCSS; Fowler et al., 2006); aged between 18 and 70; and where major changes in medication are being made, entry to the study would not occur until at least a month after stabilisation of dosage. The exclusion criteria were: a primary diagnosis of alcohol or substance dependency; organic syndrome or learning disability; a command of spoken English inadequate for engaging in therapy or the assessments; and currently having individual CBT (though previous experience of CBT was not an exclusion criterion). Recruitment into the study is summarised in the CONSORT diagram (see Fig. 2). It took place from September 2012 to March 2014 (though not continuously), with final data collection completed in May 2014. It is of note that no patient with a persecutory delusion was excluded on the basis of not endorsing at least one negative schematic belief on the BCSS.

### 2.2 Research design

The study was a randomised controlled evaluation. Patients with persecutory delusions were randomised to the CBT intervention in addition to standard care or to standard care. Randomisation was carried out in a 1:1 ratio using varying randomised permuted blocks via a sequence obtained from www.randomization.com, which was conducted by a researcher independent of the recruitment and assessment process. Random allocation followed completion of the baseline assessment. Informing patients of allocation was carried out by the trial therapists, who opened an allocation envelope. Assessments were carried out by a rater, a graduate psychologist (NE), blind to allocation. Precautionary strategies included: the therapist being encouraged to consider room use and diary arrangements in the light of potential breaks of masking; patients being reminded by the assessor not to talk about treatment allocation; and, after the initial assessment, the assessor did not look at the patients' clinical notes until the last of the ratings had been collected. If a blind was broken then another researcher within the team carried out the assessment. 28 out of 29 eight week assessments were carried out blind and all 12 week assessments were carried out blind. The assessment measures were completed at 0 weeks (baseline), 8 weeks (end of therapy), and at 12 weeks (a one month follow-up). The study had received approval from an NHS research ethics committee and was registered (ISRCTN06118265).

### 2.3 Planned interventions

The intervention was described to patients as having the aim of improving self-confidence, and was provided in six sessions to each individual over eight weeks. It was provided by clinical psychologists (DF, KP, BS, FW), with occasional support from a graduate psychologist (EC, RL) particularly for helping patients with tasks between sessions (e.g. going outside to do an activity). The strategies used were indicated in the literature to be effective at reducing negative beliefs about the self and boosting positive beliefs (and did not challenge or review the delusion itself). The strategies are described in detail in *You Can Be Happy* (Freeman and Freeman, 2012), which was provided for a number of therapy patients. The intervention is designed to provide clear and simple messages for patients to take into their day-to-day lives. There was an explicit agenda made with the patient to focus upon three areas: 1. negative thoughts about the self, 2. positive activities, and 3. positive thoughts about the self. Negative thoughts about the self were normalised, made understandable in the context of a person's experiences, and reviewed. Where voices were encouraging negative thoughts about the self the patient was helped to see this as a poor source of information and to disengage attention. Written accounts by other patients were provided. Activities were structured around the concept of five-a-day for mental well-being (Foresight Mental Capital and Wellbeing Project, 2008): connect, be active, take notice, keep learning, and give. The aim was to increase the number of these activities a patient did during the week. Positive thoughts were encouraged by reviewing the person's strengths, savouring, and keeping a diary of positive events. Positive music and pictures were also used. The patients wanted to focus on improving their self-confidence but in the instances where paranoid thoughts were brought up then the therapist would empathise with the distress and the self-confidence techniques would be framed as a way of building up strength to help deal with the difficult events occurring in the person's life. Tasks were set between sessions and there were supportive telephone calls or texts, and a graduate psychologist could visit to help with carrying out of an activity. Throughout the intervention the person's main negative self-belief and a positive self-belief were measured to monitor progress. All trial patients continued to receive standard care. Standard care was delivered according to national and local service protocols and guidelines. It usually consisted of prescription of anti-psychotic medication, visits from a community mental health worker, and regular outpatient appointments with a psychiatrist.

### 2.4 Primary outcome measures

#### 2.4.1 Brief Core Schema Scales (BCSS) (Fowler et al., 2006)

The self-report BCSS, developed with non-clinical and psychosis groups, has 24 items assessing negative and positive beliefs about the self and others each rated on a five-point scale (0–4). Higher scores reflect greater endorsement of items. The sub-scales of interest in the current study were negative beliefs about self, which contains six items (e.g. ‘I am unloved’ ‘I am worthless’ ‘I am weak’), and positive beliefs about self, which contains six items (e.g. ‘I am respected’ ‘I am valuable’ ‘I am talented’).

#### 2.4.2 Green et al. Paranoid Thoughts Scale (GPTS; Green et al., 2008)

The GPTS is a thirty-two item self-report measure of paranoid thinking, designed for both clinical and non-clinical populations. Part A assesses ideas of reference (e.g. ‘It was hard to stop thinking about people talking about me behind my back’) and Part B assesses ideas of persecution (e.g. ‘I was convinced there was a conspiracy against me’). Each item is rated on a 5-point scale. It was completed for the time period of the past fortnight. Higher scores indicate greater levels of paranoid thinking.

### 2.5 Secondary outcome measures

#### 2.5.1 Warwick-Edinburgh Mental Well-being Scale (WEMWBS) (Tennant et al., 2007)

The WEMWBS is a fourteen item scale assessing well-being over the past fortnight. Each item is rated on a 1 (none of the time) to 5 (all of the time) scale, and therefore the total score can range from 14 to 70, with higher scores indicating a greater level of well-being. Example items are: I've been feeling optimistic about the future; I've been feeling useful; I've been feeling relaxed; I've been feeling good about myself; I've been feeling confident; I've been feeling loved; and I've been feeling cheerful. The scale has high test-test reliability and criterion validity with other wellbeing scales.

#### 2.5.2 Social Comparison Scale (Allan and Gilbert, 1995)

This measure comprises eleven bipolar scales, to which we added eight further items: Inferior–superior, Incompetent–competent, unlikeable–likeable, left out–accepted, different–same, untalented–more talented, weaker–stronger, unconfident–more confident, undesirable–more desirable, unattractive–more attractive, outsider–insider, powerless–powerful, unrespected–respected, foolish–sensible, odd–normal, a failure–a success, low in self esteem–high in self esteem, a bad person–a good person, and unlike other people–like other people. Each was rated on a 0–100 scale. Cronbach's alpha for the full scale at each assessment time point was .93, .94, and .97, respectively. Higher scores indicate a more positive view of the self in relation to others.

#### 2.5.3 Psychotic Symptom Rating Scales—Delusions (PSYRATS) (Haddock et al., 1999)

The PSYRATS-Delusions scale is a six item multidimensional measure. It assesses the conviction, preoccupation, distress and disruption associated with delusions. Symptoms over the last week are rated. Higher scores indicate greater severity.

#### 2.5.4 Robson Self-Concept Questionnaire (RSQ) (Robson, 1989)

This is a 30 item self-report scale, with each item rated on a 0 (completely disagree) to 7 (completely agree) scale. Higher scores indicate higher self-esteem.

#### 2.5.5 Beck Anxiety Inventory (BAI) (Beck et al., 1988)

The BAI is a self-report 21-item, four point (0–3) scale for the assessment of anxiety over the past week. Higher scores indicate higher levels of anxiety.

#### 2.5.6 Beck Depression Inventory-II (BDI) (Beck et al., 1996)

The BDI-II is a self-report 21-item, four point scale (0–3) for the assessment of depression over the past fortnight. Higher scores indicate higher depression.

### 2.6 Adverse events

During the trial any adverse event that came to our attention was recorded. Medical notes were also examined at the end of the trial for the following events pre-specified as adverse: 1. All deaths. 2. Suicide attempts. 3. Serious violent incidents. 4. Admissions to secure units. 5. Formal complaints about therapy. Psychiatric hospital admissions were also recorded.

### 2.7 Statistical analysis

All analyses were carried out using Stata version 13 (Statacorp, 2013). All main analyses were carried out at the end of the last follow-up assessments and were based on the intention-to-treat principle. Analysis of covariance was carried out for each outcome variable, separately for 8 and 12 week assessment points. The effect of group allocation was tested, controlling for the appropriate baseline measure. At this stage of evaluation, confidence intervals for change and effect sizes are of most interest, not probability values. No formal power calculations were considered relevant. Effect sizes were calculated using Cohen's *d*, taking the estimated coefficient of treatment allocation from the ANCOVA divided by the pooled baseline standard deviation. The study target sample size was thirty patients, which would allow detection of large clinical effects.

## 3 Results

### 3.1 Basic demographic and clinical data

Table 1 provides detail of basic demographic and clinical information about the patients. The majority of patients had a clinical diagnosis of schizophrenia, they were all outpatients, and all were taking neuroleptic medication.

**Table 1:** Basic demographic and clinical data.

	Treatment group (*n* = 15)	Control group (*n* = 15)
Mean age in years (SD)	41.9 (11.5)	41.5 (13.1)
Male; female	11; 4	9; 6
Ethnicity:		
White	15	13
Other	0	2
Employment status: unemployed	13	13
Part-time employed	0	0
Full-time employed	1	1
Self employed	0	0
Retired	0	0
Student	1	1
Diagnosis: schizophrenia	12	10
Schizo-affective disorder	2	4
Delusional disorder	0	1
Psychosis NOS	1	0
Mean chlorpromazine equivalent medication dose (SD)	597.8 (355.6)	534.4 (424.7)

### 3.2 Up-take of therapy

All of the patients received the intervention. Eleven patients in the treatment group received six CBT sessions, and the intervention was provided in seven sessions for four patients. Comments from patients who had the intervention are provided in Table 2.

**Table 2:** Illustrative patient comments on the intervention.

Patient 1 “I thought it was excellent. My self-confidence has got better and I think more positively. Before, everything seemed like a really big problem and I worried a lot. I do still worry, but I tell myself I can't do anything about it so I write it down instead. I'm feeling really good at the moment.”
Patient 2 “Things are good now. I'm now able to help my wife more, it's easier to spend time with my grandchildren and I'm much more patient. I went to see my psychiatrist last week and he said I'm the best he's ever seen me. It was really helpful to talk about the personal stuff for the first time and sharing it with someone who understands. It was tough but it's gotta get bad before it gets better. When I spoke to my daughter she thought it was too tough, but something inside me told me to keep going. I'm really glad I did.”
Patient 3 “I think I am more confident and I have a slightly different bearing. The way I feel in myself and the way I am in myself. I feel more confident, a general sense of being more confident… It really helped me to reverse the balance and swing the pendulum back. It's such a change to think about strengths after years of going to the doctors and saying what's wrong. I've started giving my doctor and care co-ordinator that I meet a copy of my CV with my education, work I've done and voluntary experience on. I tell them that this is the me that you don't see when I'm well.”

### 3.3 Outcomes

The scores of the allocation groups for each assessment at each time point are displayed in Table 3. It can be seen that at posttreatment eight of the nine assessment variables showed signs of improvement with treatment, ranging from small to large effects. Five out of nine posttreatment tests reached statistical significance, with the treatment group benefiting in each case. For the primary measures there was a small effect size reduction in negative beliefs about the self and a moderate effect size reduction in paranoia, neither of which was statistically significant. For the secondary outcomes there were notable improvements in psychological well-being, positive beliefs about the self, self-esteem, social comparison, and depression. Anxiety levels were slightly lower in the control group posttreatment. No benefits of treatment were maintained at the 12 week assessment.

**Table 3:** The outcome measure scores at each assessment time.

	Treatment group			Control group							
	*n*	Mean	SD	*n*	Mean	SD	Coefficient	95% C.I.	*t*	*p*-value	Effect size
Negative self (BCSS) 0 weeks	15	11.1	6.5	15	12.5	4.3					
Negative self (BCSS) 8 weeks	15	6.4	4.7	14	8.1	3.7	− 1.3	− 4.0, 1.5	− 0.95	.353	0.24
Negative self (BCSS) 12 weeks	15	7.6	5.3	15	8.1	5.6	0.1	− 3.6, 3.9	0.07	.944	
Paranoia (GPTS) 0 weeks	15	103.7	25.2	15	117.8	24.1					
Paranoia (GPTS) 8 weeks	15	82.3	32.2	14	106.3	203	− 14.9	− 33.1, 3.3	− 1.68	.105	0.59
Paranoia (GPTS) 12 weeks	15	84.4	31.6	15	98.6	28.3	− 4.8	− 24.9, 15.2	− 0.50	.624	
Positive self (BCSS) 0 weeks	15	4.1	4.4	15	3.7	4.0					
Positive self (BCSS) 8 weeks	15	8.6	4.4	14	4.4	2.8	4.1	1.6, 6.7	3.33	.003	1.00
Positive self (BCSS) 12 weeks	15	7.3	5.4	15	6.3	4.8	0.7	− 2.5, 3.9	0.45	.657	
Social comparison (SCS) 0 weeks	15	433.8	287.3	15	408.1	193.9					
Social comparison (SCS) 8 weeks	15	903.7	326.8	14	678.1	239.1	211.8	24.3, 399.4	2.32	.028	0.88
Social comparison (SCS) 12 weeks	15	754.8	406.6	15	636.0	311.9	104.6	− 152.2, 361.3	0.84	.411	
Delusion (PSYRATS) 0 weeks	15	18.4	2.9	15	18.1	2.9					
Delusion (PSYRATS) 8 weeks	15	14.3	6.0	14	16.7	3.3	− 2.6	− 5.9, 0.7	− 1.64	.113	0.91
Delusion (PSYRATS) 12 weeks	15	15.1	4.9	15	13.7	5.4	1.0	− 2.1, 4.2	0.68	.502	
Self esteem (RSQ) 0 weeks	15	73.9	26.3	15	70.0	23.1					
Self esteem (RSQ) 8 weeks	15	99.9	21.6	14	82.9	25.6	15.2	3.0, 27.4	2.56	.017	0.62
Self esteem (RSQ) 12 weeks	15	95.3	25.9	15	83.1	30.9	9.0	− 6.5, 24.5	1.20	.242	
Well-being (WEMWBS) 0 weeks	15	30.3	6.4	15	30.1	6.6					
Well-being (WEMWBS) 8 weeks	15	41.0	7.2	14	33.5	7.3	7.4	2.2, 12.6	2.94	.007	1.16
Well-being (WEMWBS) 12 weeks	15	39.4	10.6	15	33.3	9.7	6.0	− 1.0, 13.0	1.76	.089	
Anxiety (BAI) 0 weeks	15	28.8	15.4	15	25.1	7.6					
Anxiety (BAI) 8 weeks	15	23.6	12.8	14	19.4	8.1	2.1	− 4.4, 8.7	0.67	.507	− 0.21
Anxiety (BAI) 12 weeks	15	22.9	15.3	15	21.5	7.1	− 1.8	− 6.5, 3.0	− 0.76	.452	
Depression (BDI) 0 weeks	15	32.1	11.8	15	35.1	8.4					
Depression (BDI) 8 weeks	15	21.3	9.9	14	29.1	9.0	− 6.9	− 13.3, − 0.4	− 2.19	.037	0.68
Depression (BDI) 12 weeks	15	22.9	12.6	15	27.3	9.5	− 2.3	− 9.0, 4.4	− 0.70	.492	

### 3.4 Admissions and adverse events

From medical notes it was found that one patient in each allocation group had an admission to psychiatric hospital during the trial. One patient, in the control group, made a suicide attempt. There were no deaths, serious violent incidents, admissions to forensic wards, or formal complaints about therapy.

## 4 Discussion

There is clear evidence from longitudinal and experimental studies that negative cognitions about the self are a putative causal factor in paranoia (e.g. Fowler et al., 2012, Lincoln et al., 2013, Vorontsova et al., 2013, Freeman et al., 2014a). We set-out to test this directly in patients with persecutory delusions. The interest at this stage was the immediate effects of targeting cognitions about the self. We did not try to determine which specific intervention techniques may change the mechanism. This focus on mechanism change is similar to an experimental study, though closest to a Phase II early clinical trial. The evaluation proved feasible, acceptable, and had good methodological rigour for an early stage test: assessments were blind, all patients attended all therapy sessions, and there was an exceptionally high follow-up rate. There were important gains for the patients with CBT in psychological well-being, depression, positive beliefs about the self, self-esteem, and social comparison. There were small improvements in negative beliefs about the self. As predicted there were also improvements in overall levels of paranoia and the main persistent persecutory delusions, although these did not reach statistical significance. Our view is that this indicates the potential value of targeting negative cognitions in patients with persecutory delusions.

The patients all had delusions that had persisted for a considerable period of time despite medication. Other psychotic experiences such as auditory hallucinations were very common. The therapy was very well-received: all patients attended all the sessions. Indeed there was a general readiness of patients to attend further treatment sessions. We found that positive psychology techniques and activity were most used, with much less work directly on negative views about the self. Patients appreciated a shift of focus away from their problems towards their strengths. The improvements in psychological well-being during the trial are notable and are clearly important in their own right as an outcome. This work is likely to have wider applicability to patients with psychosis. Nonetheless it may be argued that this therapy focus led to less change in negative self cognitions, which may have the closest connections to paranoid ideation. Measurement issues may also be important here, since the Brief Core Schema Scale (Fowler et al., 2006) was not developed as an outcome scale, whereas the Warwick Edinburgh Mental Well-Being Scale (Tennant et al., 2007) is well-received by patients. Further, we are struck by the gains not being maintained. Although our focus was on short-term change, we still expected gains to last. Our other brief interventions have maintained treatment gains over several months (Freeman et al., submitted for publication). Given the theoretical and research foundations for the link between negative self cognitions and paranoia, and the promise of the current study, we believe that this treatment development needs to be pursued. Our recommendations are that intervention needs to be lengthened, the targeting of negative cognitions and positive cognitions needs to be directly compared, and that an attention control condition would add strength to the judgements that can be reached. Negative self cognitions in patients with severe paranoia are likely to reflect deeply engrained self views which relate to appraisals of negative experiences over the life course, and may indeed be maintained by ongoing adverse experiences and social circumstances. How to improve negative cognitions in patients with paranoia is likely to prove an area of key interest in the future. We expect such treatment approaches in future clinical practice to be combined with interventions that target other key causal factors in paranoia (Freeman and Garety, 2014).

## Role of funding source

The study was included as part of a programme of research within a Medical Research Council (MRC) Senior Clinical Fellowship awarded to Daniel Freeman. It was therefore reviewed by the MRC before it was carried out. The funder has not had any subsequent involvement and has not seen the trial results.

## Contributors

Daniel Freeman, David Fowler and Graham Dunn designed the study. Therapy was carried out by Daniel Freeman, Katherine Pugh, Bryony Sheaves, and Felicity Waite. Recruitment and assessments were carried out by Nicole Evans, Emma Černis, and Rachel Lister. Katherine Pugh was the trial co-ordinator. Graham Dunn carried out the analysis. Daniel Freeman took responsibility for drafting the paper. All authors commented upon the final manuscript.

## Conflict of interest

None.
